# Influence of visceral fat and blood pressure on changesin blood flow velocity in non-obese individuals

**DOI:** 10.5830/CVJA-2018-001

**Published:** 2018

**Authors:** Rahman Rasyada A, Sha’ban Munirah, Azhim Azran

**Affiliations:** Department of Biotechnology, Kulliyyah of Science, International Islamic University Malaysia, Kuantan, Malaysia; Department of Physical Rehabilitation Sciences , Kulliyyahof Allied Health Sciences, International Islamic UniversityMalaysia, Kuantan, Malaysia; Department of Biomedical Sciences, Kulliyyah of AlliedHealth Sciences, International Islamic University Malaysia,Kuantan, Malaysia

**Keywords:** Doppler ultrasound, hypertension, visceral fat, nonobese, blood flow velocity

## Abstract

**Introduction:**

The aim of this study was to evaluate the impact of different visceral fat (VF) and blood pressure (BP) levels on changes in blood flow velocity (BFV) among non-obese subjects, using a cross-sectional study approach.

**Methods:**

A total of 110 putatively healthy and non-obese subjects were divided into three groups according to their level of VF and BP. Common carotid artery BFV was measured using a developed portable Doppler ultrasound measurement system.

**Results:**

The most pronounced peak systolic velocity (S1) was lower (p < 0.05) in the hypertensive group and the peak diastolic velocity (D) was significantly lower in the pre-hypertensive group than in the normotensive group. There were differences in velocity reflection and resistive indices between the hypertensive and other two BP groups. The higher VF group had significantly lower S1 and D velocities and resistive and vascular elasticity indices. By contrast, the velocity reflection index was larger in the higher VF group.

**Conclusion:**

We confirmed that there were significant differences in the BFV among non-obese subjects who differed in level of VF and BP. This study confirms that a putatively increasing VF and BP level is associated with the development of hypertension.

Obesity is one of the well-recognised cardiovascular risk factors forhypertension, dyslipidaemia and the metabolic syndrome.^1^-^3^ Bodymass index (BMI) of 30 kg/m^2^ as an indicator of obese status is usedas an important indicator of overall body fat.^4^ However, it is nowincreasingly recognised that fat distribution in specific areas can have more detrimental effects than total body fat.^2^,^3^ In particular,visceral fat (VF) is associated with hypertension, compared to other fat distributions, including lower body fat and subcutaneousfat.^2^ Previous studies have demonstrated that VF is associated withvascular disease.^4^-^6^ Increased VF accumulation contributed to thedevelopment of arteriosclerosis in a normal healthy population^7^ and coronary artery disease in non-obese patients.^6^

Numerous studies have demonstrated that Doppler spectral analysis of blood flow changes with vascular disease.^8^-^10^ Rutherhold et al. described the discriminant analysis of peak systolic (S1), peak diastolic (D) and end-diastolic (d) velocities in the diagnosis of carotid occlusive disease.^8^ The accumulation of high levels of VF contribute to greater aortic stiffness in older adults as measured by pulse-wave velocity (PWV).^10^ Furthermore, cholesterol level was found to have a correlation with mean blood flow velocity (BFV) and S1 velocity.^9^,^11^ It was suggested that patients with greater common carotid artery (CCA) plaque and intimal–medial thickness had a high velocity ratio and increased prevalence of coronary artery disease.^12^

Despite the acknowledgment that VF is associated with some haemodynamic functions, including BP and arterial PWV, the extent to which VF accumulation has an influence on BFV in CCA is not well described. Therefore, to clarify the significance of different VF levels on CCA velocities, non-obese subjects needed to be studied. In this study, we evaluated the role of the level of VF and BP on changes in BFV among non-obese subjects using a cross-sectional study approach.

## Methods

The study was performed in 110 (58 males, 52 females) putatively healthy and non-obese volunteers aged from 18 to 64 years. Overweight individuals with a body mass index (BMI) of 25 kg/ m^2^ and obese individuals with a BMI of 30 kg/m^2^, according to the World Health Organisation, were excluded from the study.^4^ The subjects had no overt chronic diseases and did not take any antihypertensive drugs, as assessed by medical history.

A written informed consent was obtained from all participants. This study was approved by the research ethics committee of the International Islamic University of Malaysia.

Three designated VF groups were based on their VF level, according to the Tanita body composition monitor: lower VF group (less than level 4), middle VF group (from level 4 to 6) and higher VF group (level 7 and above). The manual standard of the Tanita body composition monitor can track visceral fat in the body ranging from 1 to 59. A rating between 1 and 12 indicates a healthy level of visceral fat. A rating between 13 and 59 indicates an excessive level of visceral fat.^13^

For BP analysis, all subjects were further classified into three groups based on their systolic (SBP) and diastolic blood pressure (DBP) measurements: normotensive (SBP < 120 and DBP ≤ 80 mmHg), pre-hypertensive (120 ≤ SBP < 140 mmHg or 80 < DBP ≤ 89 mmHg) and hypertensive (SBP ≥ 140 and DBP > 90 mmHg).^14^

The level of VF was measured using InnerScan body composition monitors (Tanita, Japan). BMI was calculated by dividing measured body weight by the square of height (kg/ m^2^). Height and waist circumference (WC) were measured in the standing position using a stadiometer (THP-DA, Japan) and measuring tape, respectively.

The CardioChek® PA cholesterol test system was used to determine total cholesterol (TC), low-density lipoprotein (LDL) cholesterol, high-density lipoprotein (HDL) cholesterol and triglyceride (TG) levels. This device was approved by the United States Food and Drug Administration and Cholesterol Reference Method Laboratory Network.

SBP and DBP from the left brachial artery were measured in the seated position using an automatic BP monitor (Tango, SunTech Medical, USA). Mean blood pressure (MBP) was calculated from DBP + (SBP – DBP)/3.

**Fig. 1:**
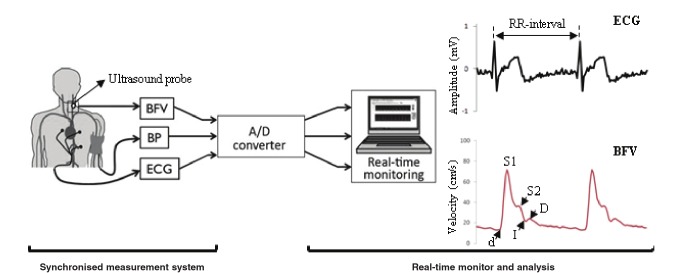
Blood flow velocity (BFV) measurement system, synchronised with electrocardiogram (ECG) and blood pressure (BP), using real-time monitoring (left). Feature points on waveform: S1: peak systolic (maximum velocity), S2: second systolic, I: incisura between systole and diastole, D: peak diastolic, and d: end-diastolic velocities (right).

The BFV measurement system was based on an application of the Doppler ultrasound technique. The portable system consisted of a probe, a Doppler signal discriminator (DSD), a transmitter at the main unit, a receiver, an analog–digital converter (A/D converter) and a computer for real-time monitoring and analysis.^15^ BFV was measured simultaneously with electrocardiogram (ECG) and BP as illustrated in Fig. 1. Measurements of ECG and BP were used as reference data.

The flow velocity (Vd) was determined from the Doppler-shift frequency (fd): V_d_ = c f_d_/(2f_0_ cosθ) where c = 1 540 m/s, the speed of acoustic waves in human tissue,f0 = 2.0 MHz, an irradiated ultrasound frequency, and θ is theDoppler insonation angle at 50 degrees.

From the Doppler shift frequency of reflected signals,low-frequency signals and harmonic noise were filtered by aband-pass filter of 0.1 to 5.0 kHz that was installed in the DSD.From the same range of frequency, BFV could be extracted.Signal data were transmitted to the receiver at a transmissionrate of 28.8 kbps and an output of ~0.5 mV/m. The data were converted into a digital signal with a sampling frequency of 10 kHz using an A/D converter, and then transferred into acomputer for real-time monitoring and signal analysis.^15^

BFV spectra were measured in the relaxed seated posture for one minute. After real-time monitoring, 30 consecutive cardiac cycles were selected from one-minute spectra to characterise the feature points of velocity waveform and calculate its indices. The waveform was extracted using a threshold method and computed using an ensemble averaging technique.

The averaged BFV waveform was used to identify velocity feature points, as shown in Fig. 1 (right side). BFV in CCA were characterised into five components: peak systolic (S1), second systolic (S2), insicura between systole and diastole (I), peak diastolic (D) and end-diastolic (d) velocities.^7^ These values were used to calculate the following velocity indices: resistive index (RI = 1 – d/S1), velocity reflection index (VRI = S2/S1 – 1) and vascular elasticity index (VEI = 1 – I/D), which were originally used by Azhim et al.^16^

## Statistical analysis

Data are expressed as mean and standard error of mean (SEM). The differences between VF groups as well as BP groups were analysed by one-way ANOVA. A p-value less than 0.05 was considered statistically significant. Statistical analyses were performed using the statistical package for the social sciences software (SPSS 21.0, USA).

## Results

Table 1 represents the differences in body mass and metabolic variables in the VF and BP groups. We found the same pattern of differences in the three designated groups of VF and BP, respectively. Participants who were older had higher VF and BP levels and greater height and weight than younger participants. BMI, WC and glucose levels were significantly greater in the higher VF and hypertensive groups. However, there were no significant differences for height, TC, HDL and LDL in all three BP groups.

**Table 1:** Subjects’ characteristics for each visceral fat and blood pressure category in the cross-sectional study

*Variable*	*Lower VF*	*Middle VF*	*Higher VF*	*Normotensive*	*Pre-hypertensive*	*Hypertensive*
Age (years)	28 ± 1	32 ± 2	49 ± 2‡§	27 ± 1	35 ± 2*	50 ± 3*†
Body mass data						
Height (cm)	161.9 ± 1.1	164.2 ± 1.3	169.5 ± 1.1‡§	162.9 ± 1.2	164.9 ± 1.1	166.5 ± 2.1
Weight (kg)	51.2 ± 0.8	60.0 ± 0.9‡	64.9 ± 1.2‡§	53.6 ± 0.9	58.0 ± 1.2*	62.9 ± 2.3*
BMI (kg/m²)	19.5 ± 0.2	22.2 ± 0.2‡	22.6 ± 0.2‡	20.2 ± 0.3	21.2 ± 0.2*	22.6 ± 0.4*†
WC (cm)	69.7 ± 0.6	77.2 ± 0.6‡	82.2 ± 0.9‡§	71.4 ± 0.7	75.7 ± 0.9*	81.8 ± 1.6*†
Metabolic variables (mg/dl)						
Glucose (mmol/l)	77.1 ± 1.9	78.5 ± 2.9	89.9 ± 3.6‡§	77.8 ± 2.1	80.3 ± 2.2	91.9 ± 5.8*†
TC (mmol/l)	195.4 ± 7.5	189.5 ± 7.0	205.8 ± 9.6	187.4 ± 6.5	206.9 ± 8.5	195.9 ± 8.9
HDL (mmol/l)	78.4 ± 3.6	80.1 ± 9.3	53.9 ± 2.9‡	75.7 ± 3.8	72.0 ± 6.5	59.8 ± 5.5
TG (mmol/l)	62.6 ± 4.8	79.8 ± 6.3	123.9 ± 15.3‡	63.7 ± 5.9	98.5 ± 10.6*	102.7 ± 18.9
LDL (mmol/l)	101.0 ± 4.9	95.0 ± 11.9	117.5 ± 6.1	100.3 ± 5.2	103.8 ± 9.2	115.6 ± 9.2

The data are presented as mean and SEM. Tukey significances: *p < 0.05 versus normotensive, †p < 0.05 versus pre-hypertensive, ‡p < 0.05 versus lower VF and §p < 0.05 versus middle VF. VF: visceral fat, BP: blood pressure; BMI: body mass index; TC: total cholesterol; HDL: high-density lipoprotein cholesterol; TG: triglycerides; LDL:low-density lipoprotein cholesterol.

As shown in Table 2, hypertensive subjects had higher VF levels compared to normotensive and pre-hypertensive subjects (p < 0.05). It is to be expected that SBP, DBP and mean BP were significantly higher in the higher VF group than in other two groups (Table 3). The most pronounced, S1 velocity, was lower (p < 0.05) in the hypertensive than the normotensive group. The D velocity was lower (p < 0.05) in the pre-hypertensive than the normotensive group.

**Table 2:** Changes in blood flow velocities and visceral fat in normotensive, pre-hypertensive and hypertensive subjects

*Variable*	*Normotensive*	*Pre-hypertensive*	*Hypertensive*	*p-value*
VF (level)	2.4 ± 0.2	5.3 ± 0.5*	8.5 ± 1*†	< 0.01
Blood flow velocities (cm/s)				
D	20.6 ± 0.7	20.3 ± 0.7	22 ± 1.4	NS
S1	100.6 ± 2.2	93.9 ± 3.4	79.4 ± 4.6*	< 0.01
S2	54.4 ± 1.9	52.6 ± 2.1	60.9 ± 2.2	NS
I	32 ± 1.3	29.7 ± 1.1	31.3 ± 1.9	NS
D	44.9 ± 1.1	41.0 ± 1.0*	39.7 ± 2.2	< 0.05
RI	0.794 ± 0.008	0.776 ± 0.009	0.719 ± 0.016*†	< 0.01
VRI	–0.453 ± 0.021	–0.412 ± 0.030	–0.215 ± 0.037*†	< 0.01
VEI	0.295 ± 0.017	0.277 ± 0.019	0.212 ± 0.021	NS

Data are presented as mean ± SEM. Significantly different: *p < 0.05 vs normotensive group; †p < 0.05 vs pre-hypertensive group. NS: not significant. VF: visceral fat; d: end-diastolic velocity; S1: peak systolic velocity; S2: second systolic velocity; I: insicura between systole and diastole; D:peak diastolic velocity; RI; VRI: velocity reflection index; VEI: vascular elasticity index.

There were differences noted in the VRI between the hypertensive and other two groups. Resistive index was significantly lower in the hypertensive than in the normotensive and pre-hypertensive groups. The other BFV waveforms, S2, d and I, showed no significant differences between the BP groups. We also found that S1, D velocities, RI and VEI indices were significantly lower in the higher VF group (p < 0.05), as shown in Table 3. By contrast, VRI was larger in the higher VF group.

**Table 3:** Effect of different levels of visceral fat on blood pressure readings and blood flow velocities

Variable	Lower VF	Middle VF	Higher VF	p-value
BP data (mmHg)				
SBP	113.1 ± 1.5	123.6 ± 2.7*	134.9 ± 3.2*†	< 0.01
DBP	68.6 ± 1.2	75.3 ± 1.8*	87.2 ± 2.6*†	< 0.01
MBP	83.5 ± 1.2	91.4 ± 1.9*	103.1 ± 2.7*†	< 0.01
Blood flow velocities (cm/s)				
D	20.5 ± 0.7	20.5 ± 1.1	20.6 ± 0.8	NS
S1	99.3 ± 2.2	98.6 ± 4.7	80.7 ± 3.2*†	< 0.01
S2	53.5 ± 1.7	54.0 ± 3.1	56.9 ± 1.7	NS
I	31.5 ± 1.2	30.3 ± 1.7	30.5 ± 1.1	NS
D	44.4 ± 1.0	42.1 ± 1.6	38.9 ± 1.2*	< 0.05
RI	0.789 ± 0.008	0.786 ± 0.012	0.740 ± 0.011*†	< 0.01
VRI	–0.449 ± 0.021	–0.426 ± 0.041	–0.278 ± 0.027*†	< 0.01
VEI	0.297 ± 0.016	0.286 ± 0.024	0.215 ± 0.014*	< 0.05

Data are presented as mean ± SEM. Significantly different: *p < 0.05 vs lower VF group; †p < 0.05 vs middle VF group.

NS: not significant, VF: visceral fat, BP: blood pressure, SBP: systolic blood pressure; DBP: diastolic blood pressure; d: end-diastolic velocity; S1: peak systolic velocity; S2: second systolic velocity; I: insicura between systole and diastole; D: peak diastolic velocity; RI; VRI: velocity reflection index; VEI:vascular elasticity index.

## Discussion

This study highlights the association between BFV changes and high VF accumulation and the development of hypertension in non-obese individuals. It is suggested that lowering VF level could reduce the incidence of hypertension as an early diseaseprevention step to improve haemodynamic function.

Fat distribution has been receiving increasing attention when evaluating the development of hypertension.^2^,^17^ Visceral fat has been demonstrated to have an association with hypertension, but not other factors, including BMI, subcutaneous fat and lower-body fat.^2^ Our study extends this analysis to emphasise the relationship between visceral hypertension and BFV of non-obese individuals.

Similar to our study, a previous study reported that individuals with essential hypertension suffered from significant accumulation of VF in the abdominal region.^2^ Our study also showed that elevated VF level leads to a significant increase in SBP, DBP and MBP (Table 3).

Significant differences in S1, D, RI, VRI and VEI were observed between the lower VF group and the other two groups. We found that S1 and D velocities decreased with increasing VF. It is to be expected that VEI in the higher VF group was significantly lower due to the significant decrease in D velocity. D is peak diastolic velocity, which increases due to vascular elastic recoil at a maximum rate.^18^ It has been reported that higher VF contributes to increased values of plaque score and β-stiffness, an index representing the stiffness of the vascular wall, which accelerates atherosclerosis.^5^ Stiffness of the artery is indicated by its elastic properties. This observation is consistent with our finding in which the higher VF group had significantly lower D and VEI values than those of the lower VF group.

Similar to the higher VF group, the S1 and D velocity peaks declined in the hypertensive group (Table 2). This might have been due to the fact that arteries stiffen with age,^14^,^19^ since both groups were older and had VF. Furthermore, the thickening of the arterial wall, which is caused by VF, could induce high blood pressure.^20^

VRI has been demonstrated to be a good index of cardiovascular risk in hypertensive patients compared to control subjects.^10^,^21^ VRI is linked to reflection characteristics of velocity.^14^ This study showed that there were significant differences between the hypertensive and normotensive groups in both VRI and RI values. The RI is a well-recognised index for quantifying changes in CCA.^20^,^22^. This index is widely used as an indicator of peripheral vascular resistance.^20^ A previous study reported that RI was higher in severe internal carotid artery stenosis, compared to a normal carotid artery of patients.^22^

Limitations of this study are that we used a cross-sectional approach only, and the three designated VF groups did not consider gender and age differences. In our current setting, it was difficult to find a large number of subjects of the same age with different levels of VF. Further interventional studies on welldiscriminated groups are required to show a distinction between cause and effect among non-obese subjects.

## Conclusion

We found significant differences in BFV among non-obese subjects with different levels of VF and BP. The study also supports the alleged association between increasing VF and BP levels and the development of hypertension.
